# Meta-GWAS and Meta-Analysis of Exome Array Studies Do Not Reveal Genetic Determinants of Serum Hepcidin

**DOI:** 10.1371/journal.pone.0166628

**Published:** 2016-11-15

**Authors:** Tessel E. Galesloot, Niek Verweij, Michela Traglia, Caterina Barbieri, Freerk van Dijk, Anneke J. Geurts-Moespot, Domenico Girelli, Lambertus A. L. M. Kiemeney, Fred C. G. J. Sweep, Morris A. Swertz, Peter van der Meer, Clara Camaschella, Daniela Toniolo, Sita H. Vermeulen, Pim van der Harst, Dorine W. Swinkels

**Affiliations:** 1 Radboud university medical center, Radboud Institute for Health Sciences, Nijmegen, The Netherlands; 2 University of Groningen, University Medical Center Groningen, Department of Cardiology, Groningen, The Netherlands; 3 Division of Genetics and Cell Biology, San Raffaele Scientific Institute, Milano, Italy; 4 Department of Medical, Surgical and Health Sciences, University of Trieste, Trieste, Italy; 5 University of Groningen, University Medical Center Groningen, Genomics Coordination Center, Groningen, The Netherlands; 6 University of Groningen, University Medical Center Groningen, Department of Genetics, Groningen, The Netherlands; 7 Radboud university medical center, Radboud Institute for Molecular Life Sciences, Nijmegen, The Netherlands; 8 Hepcidinanalysis.com, Department of Laboratory Medicine, Radboud university medical center, Nijmegen, The Netherlands; 9 Department of Medicine, Section of Internal Medicine, University of Verona, Verona, Italy; 10 Vita-Salute University, Milan, Italy; Lady Davis Institute for Medical Research, CANADA

## Abstract

Serum hepcidin concentration is regulated by iron status, inflammation, erythropoiesis and numerous other factors, but underlying processes are incompletely understood. We studied the association of common and rare single nucleotide variants (SNVs) with serum hepcidin in one Italian study and two large Dutch population-based studies. We genotyped common SNVs with genome-wide association study (GWAS) arrays and subsequently performed imputation using the 1000 Genomes reference panel. Cohort-specific GWAS were performed for log-transformed serum hepcidin, adjusted for age and gender, and results were combined in a fixed-effects meta-analysis (total N 6,096). Six top SNVs (p<5x10^-6^) were genotyped in 3,821 additional samples, but associations were not replicated. Furthermore, we meta-analyzed cohort-specific exome array association results of rare SNVs with serum hepcidin that were available for two of the three cohorts (total N 3,226), but no exome-wide significant signal (p<1.4x10^-6^) was identified. Gene-based meta-analyses revealed 19 genes that showed significant association with hepcidin. Our results suggest the absence of common SNVs and rare exonic SNVs explaining a large proportion of phenotypic variation in serum hepcidin. We recommend extension of our study once additional substantial cohorts with hepcidin measurements, GWAS and/or exome array data become available in order to increase power to identify variants that explain a smaller proportion of hepcidin variation. In addition, we encourage follow-up of the potentially interesting genes that resulted from the gene-based analysis of low-frequency and rare variants.

## Introduction

Iron is an essential trace element for fundamental metabolic processes in humans [1, 2]. Iron deficiency limits hemoglobin synthesis and leads to anemia, whereas an excess of free iron is toxic because it catalyzes the production of free radicals resulting in tissue damage [1, 2]. In addition, iron imbalances have been associated with other diseases, *e*.*g*. diabetes mellitus [3, 4], inflammation [5] and diseases of aging [6]. Hence, the iron balance in the human body is tightly controlled, with the peptide hormone hepcidin as key regulator of systemic iron homeostasis [7]. Hepcidin controls the absorption, storage and tissue distribution of iron by binding to the cellular iron exporter ferroportin and inducing its internalization and degradation [8]. In this way, hepcidin regulates the uptake of dietary iron from the intestine and the release of iron from macrophages involved in recycling of iron from senescent erythrocytes [7].

In the last few years, several genome-wide association studies (GWAS) have revealed genetic variants associated with iron status in the general population, including common variants in the hereditary hemochromatosis gene (*HFE*), transferrin gene (*TF*), transferrin receptor and transferrin receptor 2 gene (*TFRC*, *TFR2*), solute carrier family 40 member 1 gene (*SLC40A1*), and transmembrane serine protease 6 gene (*TMPRSS6*) [9–14]. On the contrary, little is known about genetic determinants of hepcidin. Mutations in hepcidin antimicrobial peptide (*HAMP*), the hepcidin encoding gene, lead to strongly decreased hepcidin levels and a severe juvenile form of the iron storage disorder hereditary hemochromatosis (HH), but *HAMP* mutations are very rare [15]. In addition, mutations in *HFE*, *TFR2* and *TMPRSS6* have been related to hepcidin expression [15–20]. Furthermore, a single GWAS for serum hepcidin has been published [21]. This study among 1,657 family members from the Val Borbera genetic isolate was however underpowered to identify genome-wide significant associations [21]. Here, we aimed to identify genetic determinants of serum hepcidin in a larger set of individuals from three large cohorts in order to unravel potential new pathways involved in hepcidin regulation. We also studied the ratios of hepcidin to ferritin (hepcidin/ferritin) and hepcidin to transferrin saturation (TS) (hepcidin/TS) given the known dependence of hepcidin on stored iron and circulating iron, respectively [1, 2].

## Materials and Methods

### Study populations

We included three cohorts in our study: the Nijmegen Biomedical Study (NBS) (Nijmegen, The Netherlands) [22], Prevention of REnal and Vascular ENd-stage Disease (PREVEND) (Groningen, The Netherlands) [23] and Val Borbera (VB) (Milan, Italy) [24, 25] (S1 Table). Blood samples for DNA isolation and biochemical measurements were obtained fasting in the morning for PREVEND and VB, whereas blood samples were not fasting and sampled throughout the day between 8 AM and 9 PM for NBS. All three studies were approved by appropriate ethical committees (PREVEND: local medical ethics committee; NBS: Radboud university medical center Institutional Review Board; VB: institutional review boards of San Raffaele Hospital in Milan and by the Regione Piemonte ethical committee), and all participants gave informed consent.

### Laboratory methods

Serum hepcidin concentration was measured with a competitive enzyme-linked immunosorbent assay in NBS and PREVEND samples as described before [26, 27]. In VB samples, serum hepcidin was measured with a validated mass spectrometry based method as described before [21]. Serum ferritin, iron, transferrin, TS and C-reactive protein (CRP) were measured according to standard methods. See S2 Table for details. Phenotype information (median and 5^th^-95^th^ percentiles) is presented in S3 Table.

### Genotyping

All three cohorts were genotyped with a GWAS-chip: PREVEND with the Illumina Cyto SNP12 v2, NBS with the Illumina HumanHap370CNV-Duo BeadChip, and VB with the Illumina HumanHap370CNV-Quad BeadChip v3. Standard quality checks were performed (filters for sample yield, SNV yield, MAF and HWE) and data were imputed to increase SNV density and harmonize SNV data over the cohorts using 1000 Genomes phase 1 version 3 as reference panel (S4 Table). Quality control also included evaluation of population stratification. For PREVEND, principal component analysis was used and samples with a Z-score>3 for the first five principal components were excluded. For NBS, Structure analysis was used and samples with less than 89% Caucasian ancestry were excluded. For VB, principal components were used in the analysis to adjust for potential population stratification.

### Genome-wide association analysis

GWAS were performed in each cohort separately according to a set protocol. Analyses were performed for all individuals (PREVEND: N = 2,902; NBS: N = 1,819; VB: N = 1,480), and also for a subset (PREVEND: N = 2,695; NBS: N = 1,495; VB: N = 1,206) from which individuals with ferritin <30 ng/mL and CRP ≥10 mg/L were excluded as to remove individuals with iron deficiency and clinical inflammation [28], respectively, since both of these acquired conditions are associated with altered iron stores and altered iron transport [1,2]. Hepcidin and the ratios hepcidin/ferritin and hepcidin/TS were log-transformed and thereafter adjusted for age and squared age, independent determinants of serum hepcidin [21, 26], separately for males and females. For NBS, blood sampling was performed throughout the day, and therefore time of blood sampling was used as an additional covariate to account for the circadian rhythm of hepcidin [29] (three categories: before 12 PM, between 12 and 5 PM and after 5 PM in line with previously reported hepcidin concentration patterns throughout the day [30, 31]). Sex‐specific residuals were calculated and merged into one variable. Outliers, defined as values that differed more than four times the SD from the mean, were excluded (Nmax = 12). For PREVEND and NBS, the association between the single nucleotide variants and the trait was tested by linear regression using genotype probabilities and an additive model on the standardized residuals (Z score). For VB, a linear mixed model was used with a kinship (relatedness) matrix to account for the relatedness in this sample [32], also using genotype probabilities and an additive model on the standardized residuals (Z score).

### Meta-analysis

The GWAS results from the three cohorts were combined in a fixed-effects meta-analysis using the software package METAL [33]. The standard-error based approach was used, which weighs effect size estimates using the inverse of the corresponding standard errors. Variants with a minor allele frequency <1% and a poor imputation quality (SNPtest info value or MACH RSQR <0.4) were excluded prior to the meta-analysis. To adjust for potential residual population stratification, we applied genomic control correction to the individual cohorts (genomic inflation factors [lambdas] for GWAS results ranged from 0.989 to 1.015 in the three cohorts, indicating a negligible amount of population stratification) [34]. A heterogeneity analysis was performed to test whether observed effect sizes were homogeneous across cohorts. Resulting betas express the change in log-transformed hepcidin (or the ratios) that can be attributed to each copy of the effect allele (additive model).

### Replication

Our financial budget allowed us to genotype six SNVs with single SNP assays in all additional independent samples that were available for PREVEND (N = 2,876) and NBS (N = 1018). For the VB cohort, no additional samples were available. Single-SNP genotyping in PREVEND samples was performed by KBiosciences (KBiosciences, Herts, UK) utilizing the SNPline system. Single-SNP genotyping in NBS samples was carried out by deCODE Genetics using the Centaurus (Nanogen) platform [35]. The quality of each Centaurus SNP assay was evaluated by genotyping HapMap CEU samples with each assay and comparing the results with the HapMap data. All assays were reliable, as the mismatch rates were all <0.5%. Association analyses were performed using the same strategy as for the discovery meta-GWAS: cohort-specific association analyses and subsequent combination of summary statistics in a fixed-effects meta-analysis. We also meta-analyzed these replication results together with the discovery meta-analysis results.

### Gene-based analysis

We performed gene-based analysis on SNV association P-values from the meta-analysis of discovery samples using VEGAS2 [36, 37]. Statistical significance of gene-based analysis results was based on Bonferroni correction of testing ~21,000 genes (P<2.4x10^-6^).

### DEPICT

Data-driven Expression Prioritized Integration for Complex Traits (DEPICT, “v1 beta version rel194 for 1KG imputed”) was applied to the meta-GWAS discovery results to highlight enriched pathways and identify tissues/cell types in which genes from associated loci are highly expressed [38]. Meta-GWAS association results with p<1x10^-5^ were pruned using PLINK v1.09 to obtain independent SNVs (‘—clump-kb 500—clump-p1 1e-05—clump-r2 0.1’) using the CEU (Utah Residents [CEPH] with Northern and Western Ancestry), GBR (British in England and Scotland) and TSI (Toscani in Italy) 1000 Genomes populations as reference to obtain the correlation structure of the SNVs. These pruned data were used as input for the DEPICT analysis using default settings. Details of the DEPICT analysis can be found in S1 File.

### Exome array association analysis

Exome array data measured with the Illumina HumanExome BeadChip were available for both NBS and VB. Genotype data were called with the default genotype caller in Genome Studio and uncalled genotypes were recalled using the zCall algorithm, which is specifically designed for calling rare variants [39]. Filters for sample yield, SNV yield, and HWE were applied, resulting in a total N of1,753 for NBS and 1,473 for VB after QC (S5 Table). Phenotype information for the exome array samples is given in S6 Table. Analyses were performed using the phenotypes as described under ‘Genome-wide association analysis’. Single variant analyses were performed to obtain cohort specific score statistics and their covariance matrix (RvTests software version 20150629 [http://zhanxw.github.io/rvtests/]). For VB, a kinship matrix was included in the analyses to account for relatedness. Cohort specific results were meta-analyzed in RareMETAL software version 4.13.8 (http://genome.sph.umich.edu/wiki/RAREMETAL_Documentation) on single variant level and in a gene-level test [sequence kernel association test (SKAT)] [40]; only non-synonymous, splice site and stop variants with a MAF ≤5% were included in the gene-level test.

Single variant results were filtered for a pooled minor allele frequency between 0.001 and 0.05 to prevent repeated analysis of the common variants and at the same time reduce the number of statistical tests. The lower bound frequency of 0.001 corresponds to a minor allele count of 6 for the whole cohort with exome array data (N up to 3,226), and 5 for the subset (N up to 2,623).

SKAT meta-analysis in RareMETAL presents an exact and approximate p-value in the output. We used the exact test called “Davies”, which computes the p-value by inverting the characteristic function of the mixture chisq, which is often used as the default in statistical analysis packages.

The statistical significance of single variant and gene-based exome array meta-analysis results was based on Bonferroni correction of testing ~37,000 variants (p< 1.4 x10^-6^) and ~14,000 genes (p< 3.6 x10^-6^), respectively.

### Power calculation

Power analysis were performed using GWAPower Detection V1.1 [41]. Effective sample size of the VB cohort to take into account relatedness was determined using Greffa software [42] based on a pairwise kinship coefficient <0.0625, resulting in an effective sample size of N = 714.

## Results

Meta-analysis of GWAS (total N 6,096) revealed two loci that were genome-wide significantly associated (p<5x10^-8^) with serum hepcidin (Table 1): one on chromosome 10 in all individuals (rs118031191, nearest gene *FOXI2*) and one on chromosome 2 in the subset (four SNVs in *EML6* with lead SNV rs354202). S1 and S2 Figs show Manhattan and QQ plots for the meta-GWAS for hepcidin in all individuals and in the subset, respectively. S3 and S4 Figs show the regional association plots for rs118031191 and rs354202, respectively. We also performed conditional analysis for the chromosome 2 locus in NBS data by adjusting for lead SNV rs354202 to investigate whether the additional signals at this locus identified in the discovery analysis were independent from rs354202. Associations disappeared upon adjustment for this SNV, revealing that all other signals at the chromosome 2 locus were driven by rs354202 (S5 and S6 Figs). No novel significant loci were found for the ratios hepcidin/ferritin and hepcidin/TS (Manhattan and QQ plots in S7–S10 Figs), but previously identified associations were confirmed: for the hepcidin/ferritin ratio we observed genome-wide significant associations with variation in *HFE* and *TMPRSS6* (S7 Table), and for hepcidin/TS with variation in *TMPRSS6*, but not with *HFE* (S8 Table).

**Table 1:** Top hits (p-value<1x10^-6^) for hepcidin in all individuals and in the subset based on meta-analysis of three GWAS. Genome-wide significant SNVs (p-value<5x10^-8^) are indicated in bold.

						All						Subset					
SNV	CHR	BP (Build 37)	In gene/nearest gene	A1*	A2	Freq A1	Beta	SE	p	Direction^#^	N^†^	Freq A1	Beta	SE	p	Direction^#^	N
rs12477708	2	54905508	EML6	A	G	0.10	0.14	0.03	2.83E-05	+++	6096	0.10	0.19	0.04	1.69E-07	+++	5051
rs80098840	2	54918152	EML6	A	G	0.89	-0.14	0.03	5.73E-06	---	6096	0.89	-0.18	0.03	1.51E-07	---	5051
**rs76949049**	**2**	**54965697**	**EML6**	**T**	**C**	**0.10**	**0.16**	**0.03**	**1.14E-06**	**+++**	**6096**	**0.10**	**0.20**	**0.04**	**2.15E-08**	**+++**	**5051**
**rs354202**	**2**	**54970943**	**EML6**	**A**	**G**	**0.90**	**-0.17**	**0.03**	**7.02E-08**	**---**	**6096**	**0.90**	**-0.20**	**0.03**	**1.21E-08**	**---**	**5051**
rs354204	2	54971385	EML6	A	G	0.86	-0.12	0.03	4.92E-06	---	6096	0.86	-0.16	0.03	5.30E-08	---	5051
rs9973793	2	54998516	EML6	T	C	0.13	0.12	0.03	2.11E-05	+++	6096	0.13	0.16	0.03	2.84E-07	+++	5051
**rs2033823**	**2**	**55057740**	**EML6**	**T**	**C**	**0.90**	**-0.13**	**0.03**	**1.11E-05**	**---**	**6096**	**0.90**	**-0.19**	**0.03**	**2.64E-08**	**---**	**5051**
**rs13420395**	**2**	**55058720**	**EML6**	**A**	**G**	**0.10**	**0.13**	**0.03**	**1.32E-05**	**+++**	**6096**	**0.10**	**0.19**	**0.03**	**2.79E-08**	**+++**	**5051**
rs7592363	2	55060479	EML6	T	C	0.10	0.13	0.03	1.39E-05	+++	6096	0.10	0.18	0.03	6.11E-08	+++	5051
rs6747033	2	55061294	EML6	C	G	0.89	-0.14	0.03	6.65E-06	---	6096	0.89	-0.18	0.03	1.10E-07	---	5051
rs56281245	5	145007639	PRELID2^‡^	T	C	0.95	0.17	0.05	6.00E-04	+++	6096	0.95	0.26	0.05	6.66E-07	+++	5051
rs11388147	7	71647721	CALN1	G	GA	0.27	-0.15	0.03	9.63E-07	?--	3279	0.26	-0.13	0.03	7.98E-05	?--	2695
**rs118031191**	**10**	**129582469**	**FOXI2**^‡^	**A**	**G**	**0.03**	**-0.38**	**0.07**	**1.59E-08**	**--**	**6096**	**0.03**	**-0.33**	**0.08**	**1.41E-05**	**---**	**5051**
rs12289793	11	21348000	NELL1	A	G	0.79	0.10	0.03	1.60E-04	+++	6096	0.79	0.14	0.03	9.91E-07	+++	5051
rs117568227	12	66447376	LLPH^‡^^,^^$^	A	G	0.01	-1.03	0.21	7.82E-07	?-?	1479	0.01	-1.19	0.24	5.85E-07	?-?	1206
rs150188223	13	42844491	AKAP11	T	C	0.01	-0.57	0.18	1.41E-03	??-	1800	0.01	-1.00	0.20	5.10E-07	??-	1489
rs141939445	20	36896818	KIAA1755^‡^^,^^$^	T	C	0.99	0.71	0.16	6.87E-06	?++	3279	0.99	0.91	0.18	1.98E-07	?++	2695

Analyses were performed for all individuals with a hepcidin measurement above the detection limit of the hepcidin assay.The betas express the change in log-transformed hepcidin that can be attributed to each copy of allele 1 (additive model). A indicates allele; BP, base pair position; CHR, chromosome; Freq, frequency; N, number; SE, standard error; SNV, single nucleotide variant.

*A1 is the effect allele in the association analysis.

^#^Order of direction: PREVEND, VB, NBS. A question mark (?) indicates that the variant had a minor allele frequency <1%, and/or a SNPtest info value or MACH RSQR <0.4, and/or was not imputed in a cohort.

†The number of individuals per SNV that is included in the analysis varies with the amount of cohorts for which the SNV was available for analysis.

^‡^These SNVs lie in intergenic regions.

^$^Closer than LLPH lies RNA, 5S ribosomal pseudogene 362 (RNA5SP362).

Gene-based analysis (S9 Table for results with p<1x10^-2^) did only show significant association for hepcidin/ferritin with *HFE* (all p = 7.2x10^-7^; subset p = 8.2x10^-7^). No significant enriched gene sets or tissues were identified for all traits (S10 and S11 Tables).

Six SNVs were brought forward to replication: rs354202, rs118031191, rs56281245 and rs12289793 were selected based on p<1x10^-6^ for association with hepcidin, and rs1835473 and rs12441903 were additionally selected based on hepcidin association p-values close to 1x10^-6^, location inside genes, and MAF >10%. All SNVs selected for replication had a non-significant p-value for the heterogeneity test (p>0.05). Replication analysis (N 3,821) revealed no significant associations at p = 0.05 (Table 2).

**Table 2:** Results of the replication analyses and discovery and replication combined.

						Replication					Discovery + Replication				
SNV	Population	A1*	A2	Freq A1 PREVEND	Freq A1 NBS	Beta	SE	p	Direction^#^	N	Beta	SE	p	Direction†	N
rs12289793	All	A	G	0.78	0.72	0.02	0.03	0.38	++	3770	0.06	0.02	7.01E-04	+++	9866
	Subset	A	G	0.78	0.74	0.03	0.03	0.31	++	3072	0.09	0.02	1.49E-05	+++	8123
rs1835473	All	A	G	0.68	0.70	0.03	0.02	0.32	++	3754	0.07	0.02	1.48E-05	+++	9850
	Subset	A	G	0.68	0.70	0.02	0.03	0.49	+-	3059	0.05	0.02	2.27E-03	++-	8110
rs56281245	All	T	C	0.95	0.95	0.06	0.05	0.24	++	3798	0.12	0.04	8.40E-04	+++	9894
	Subset	T	C	0.95	0.96	0.04	0.06	0.56	++	3092	0.16	0.04	3.83E-05	+++	8143
rs118031191	All	A	G	0.03	0.03	0.00	0.07	1.00	-+	3821	-0.18	0.05	9.12E-05	--+	9917
	Subset	A	G	0.03	0.03	0.00	0.07	0.96	-+	3115	-0.16	0.05	2.60E-03	--+	8166
rs12441903	All	A	G	0.89	0.87	-0.04	0.04	0.33	--	3816	-0.10	0.02	3.13E-05	---	9912
	Subset	A	G	0.89	0.87	-0.03	0.04	0.41	--	3108	-0.10	0.03	6.29E-05	---	8159
rs354202‡	All	A	G	0.89	0.89	-0.03	0.04	0.39	+-	3810	-0.11	0.02	3.32E-06	-+-	9906
	Subset	A	G	0.90	0.89	0.00	0.04	0.92	-+	3109	-0.12	0.03	9.24E-06	—+	8160

A indicates allele; BP, base pair position; Freq, frequency; N, number; SE, standard error; SNV, single nucleotide variant. Association analysis were performed using the same strategy as for the discover meta-GWAS: cohort-specific association analyses and subsequent combination of summary statistics in a meta-analysis using only replication samples (Replication) and the discovery meta-analysis and replication samples combined (Discovery + Replication). The betas express the change in log-transformed hepcidin that can be attributed to each copy of allele 1 (additive model). HWE p-values in PREVEND and NBS, respectively, were for rs12289793: p = 0.71 and 0.89; for rs1835473 p = 0.001 and 0.90; for rs56281245 p = 0.12 and 0.90; for rs118031191 p = 0.04 and 0.29; for rs12441903 p = 0.04 and 0.95; and for rs354202 p = 0.051 and 0.45.

*A1 is the effect allele in the association analysis.

^#^Order of direction: NBS, PREVEND.

†Order of direction: discovery meta-analysis, NBS, PREVEND.

‡In PREVEND, a proxy of rs354202 was measured (rs76949049; r^2^ with rs354202 = 1).

Meta-analysis on single variant level of exome array variants (total N 3,226) revealed no significantly associated SNVs for hepcidin (S12 Table presents results with p<1x10^-3^). The most significant signal was located in *PCDHB1* (exm485355, p = 4.4x10^-5^) for all individuals and in *PTPN13* for the subset (exm411306, p = 2.1x10^-5^). Also for hepcidin/TS no significant signals were observed, but for hepcidin/ferritin we identified two signals that reached exome-wide significance: exm470499 in *WDR36* (all; p = 9.9x10^-7^) and exm162358 in *MTR* (subset; p = 2.7x10^-8^); variants close to and in these genes have previously been associated with allergy [43] and homocysteine levels [44], respectively. Gene-based meta-analyses (S13 Table) revealed eight genes that showed significant association with hepcidin in all individuals, and 11 in the subset, but none of these genes overlapped. For hepcidin/ferritin, 10 and nine genes showed significant association in all individuals and in the subset, respectively, with one overlapping gene, namely *PAPSS1*. This gene was described as a candidate gene for telomere length based on a GWAS [45]. Previously, elevated iron phenotype was associated with shortened telomeres [46], indicating a potential link between this gene and systemic iron homeostasis. For hepcidin/TS, nine and 16 genes were significant in all individuals and in the subset, respectively, but none overlapped.

## Discussion

This is the first meta-analysis of GWAS and exome array results for serum hepcidin. The fact that our meta-analysis revealed no SNVs that were significantly associated with serum hepcidin suggests that there are no common, low-frequency or rare variants that explain a large proportion of phenotypic variation in serum hepcidin. Indeed, with our meta-analysis of common variants (N 6,096, effective sample size N 5,331) we had 80.0% and 97.8% chance of detecting (at alpha 5x10^-8^) a SNV that explains 0.71% and 1.00% of hepcidin variance, respectively. For our meta-analysis of exome array results (N 3,226, effective N 2,467) we had 80.0% and 99.2% chance of detecting (at alpha 1.4x10^-6^) a SNV explaining 1.22% and 2.00% of hepcidin variance, respectively. For comparison, the well-known iron-related SNVs rs1800562 in *HFE* and rs855791 in *TMPRSS6* explain ~1% of serum iron variation. In addition, (narrow-sense) heritability of hepcidin was previously estimated to be 9.8% but non-significant (p>0.05) in the VB population and genome-wide SNP explained variance adjusted for age and gender was estimated at ~37% (SE~20%) in the NBS (data not shown). Overall, this suggests that a large part of hepcidin variability is caused by variation in environmental factors, *e*.*g*. inflammation, body-mass index and body iron stores (dietary intake and blood losses).

Serum hepcidin was not associated with common variants in *HFE* and *TMPRSS6*, as we have previously shown in independent studies both in the VB and NBS population [21, 47]. Now, we have also shown that low-frequency or rare variants in these genes do not seem to contribute, neither at single variant level nor at gene level. We confirmed previously reported associations for rs1800562 in *HFE* and rs855791 in *TMPRSS6* with the ratio hepcidin/ferritin for the VB and NBS population [21, 47]. As expected, we further substantiated these associations here and found an even stronger signal. The association signal of the ratio hepcidin/TS with common variants in *HFE* and *TMPRSS6* was less strong. Of note, the association of rs1800562 in *HFE* with the ratio hepcidin/TS, previously found in the NBS [47], disappeared upon meta-analysis of results of NBS, PREVEND and VB in all individuals (p = 0.13), but still showed a weak signal in the subset (p = 3.7Ex10^-4^). Also rs855791 in *TMPRSS6* showed a stronger signal for association with the ratio hepcidin/TS in the subset compared to analysis based on all individuals. The stronger signal of rs1800562 and rs855791 with the ratio hepcidin/ferritin compared to the ratio hepcidin/TS indicates that these SNVs have a larger influence on hepcidin response to body iron stores than on hepcidin response to circulating iron.

There are several reasons that can cause failure of identifying a true association signal [48, 49], *e*.*g*. issues of heterogeneity between studies, such as variability in outcome measurement or statistical analysis, or bias due to population stratification. We took those issues into account by using a set analysis protocol for cohort-specific analysis, testing for heterogeneity for the top hits, and adjusting for population stratification in each cohort next to application of genomic control adjustment. Two different hepcidin assays were applied in this study, but correlation between these assays was high as measured in international *round robins* (sample send-out studies) for hepcidin assay harmonization (DWS and DG, unpublished findings). In addition, we prevented bias in SNV-hepcidin associations due to differences in hepcidin assays by first performing cohort-specific GWAS and thereafter combining results in a meta-analysis. However, it is important to realize that there are additional reasons that may have masked true association results in our study, *e*.*g*. residual confounding due to population stratification, presence of comorbidities that associate with serum hepcidin levels, incomplete adjustment for relatedness in the VB cohort, genetic or environmental interactions [48, 49], or high biological variation in serum hepcidin levels [29, 30, 31, 50].

In conclusion, our results indicate that there are no common SNVs that explain more than 1% and no low-frequency and rare SNVs that explain more than 2% of phenotypic hepcidin variation. We recommend extension of our study once additional substantial cohorts with hepcidin measurements, GWAS and/or exome array data become available in order to increase power to identify variants that explain a smaller proportion of hepcidin variation. In addition, we encourage follow-up of the potentially interesting genes that resulted from the gene-based analysis of low-frequency and rare variants with candidate gene, fine mapping and functional studies to increase the level of evidence for association and obtain insight into the underlying mechanism of action.

## Supporting Information

S1 Table Cohort information and acknowledgments. (DOCX)

S2 Table Laboratory measurements. (DOCX)

S3 Table Phenotype information [median (P5-P95)] of the samples included in the meta-GWAS. (DOCX)

S4 Table Information about genotyping, imputation and quality control of the cohort-specific GWAS. (DOCX)

S5 Table Information about genotyping and quality control of the cohort-specific exome array analysis. (DOCX)

S6 Table Phenotype information of the samples included in the exome array analyses [median (5th percentile-95th percentile)]. (DOCX)

S7 Table Top hits (p-value < 1E-06) for hepcidin/ferritin in all individuals and in the subset. (XLSX)

S8 Table Top hits (p-value < 1E-06) for hepcidin/TS in all individuals and in the subset. (XLSX)

S9 Table VEGAS results. (XLSX)

S10 Table DEPICT results tissue enrichment. (XLS)

S11 Table DEPICT results gene set enrichment. (XLS)

S12 Table Exome array single variants results. (XLSX)

S13 Table Exome array gene-based (SKAT) results. (XLSX)

S1 Fig Manhattan plot and QQ plot for the meta-analysis results for hepcidin in all individuals. (DOCX)

S2 Fig Manhattan plot and QQ plot for the meta-analysis results for hepcidin in the subset. (DOCX)

S3 Fig Regional association plot for rs118031191 with serum hepcidin in all individuals. (DOCX)

S4 Fig Regional association plot for rs354202 with serum hepcidin in the subset. (DOCX)

S5 Fig Regional association plot for the chromosome 2 locus with serum hepcidin conditioned on rs354202 in all individuals (NBS data only). (DOCX)

S6 Fig Regional association plot for the chromosome 2 locus with serum hepcidin conditioned on rs354202 in the subset (NBS data only). (DOCX)

S7 Fig Manhattan plot and QQ plot for the meta-analysis results for the ratio hepcidin/ferritin in all individuals. (DOCX)

S8 Fig Manhattan plot and QQ plot for the meta-analysis results for the ratio hepcidin/ferritin in the subset. (DOCX)

S9 Fig Manhattan plot and QQ plot for the meta-analysis results for the ratio hepcidin/TS in all individuals. (DOCX)

S10 Fig Manhattan plot and QQ plot for the meta-analysis results for the ratio hepcidin/TS in the subset. (DOCX)

S1 File Supplemental Methods. (DOCX)
