# Supplementary material for: Meta-GWAS and Meta-Analysis of Exome Array Studies Do Not Reveal Genetic Determinants of Serum Hepcidin
Source: PLoS One. 2016 Nov 15;11(11):e0166628. doi: 10.1371/journal.pone.0166628 (PMC5112847; doi:10.1371/journal.pone.0166628)
Supplement: S1 Table — (DOCX) [file pone.0166628.s001.docx]

**S1 Table.** Cohort information and acknowledgements.

| **Cohort name** | **Cohort description and references** | **Financial support** | **Acknowledgements** |
| --- | --- | --- | --- |
| Nijmegen Biomedical Study (NBS) | The Nijmegen Biomedical Study (NBS; http://www.nijmegenbiomedischestudie.nl) is a population-based survey conducted by the Department for Health Evidence and the Department of Laboratory Medicine of the Radboud University Medical Centre, Nijmegen, The Netherlands. The study has been described before (Hoogendoorn et al., Clin Chem 2006)). Briefly, in 2002, 22,451 age and sex-stratified randomly selected adult inhabitants of Nijmegen, a city located in the eastern part of the Netherlands, received an invitation to fill out a postal questionnaire (QN) including questions about lifestyle, health status, and medical history, and to donate a blood sample for DNA isolation and biochemical studies. A total of 9350 (43%) persons filled out the QN, of which 6468 (69%) donated blood samples. A second, third and fourth questionnaire were sent out in 2005, 2008 and 2012, respectively. Approval to conduct the NBS was obtained from the Radboud university medical center Institutional Review Board. All participants gave written informed consent for participation in the NBS. For this study we used the subset of 1819 NBS participants that was selected to serve as controls in GWAS (Kiemeney et al., Nat Genet 2008). | This work was sponsored by the Stichting Nationale Computerfaciliteiten (National Computing Facilities Foundation, NCF) for the use of supercomputer facilities, with financial support from the Nederlandse Organisatie voor Wetenschappelijk Onderzoek (Netherlands Organization for Scientific Research, NWO). | The Nijmegen Biomedical Study is a population-based survey conducted at the Department for Health Evidence, and the Department of Laboratory Medicine of the Radboud university medical center. Principal investigators of the Nijmegen Biomedical Study are L.A.L.M. Kiemeney, A.L.M. Verbeek, D.W. Swinkels and B. Franke.  We thank Doorlène van Tienoven for performing the serum hepcidin measurements. |

| The Prevention of REnal and Vascular ENd stage Disease study (PREVEND) | The PREVEND Study is a prospective, observational cohort study, focussed to assess the impact of elevated urinary albumin loss in non-diabetic subjects on future cardiovascular and renal disease. PREVEND is an acronym for Prevention of REnal and Vascular ENd-stage Disease. This study started with a population survey on the prevalence of micro-albuminuria and generation of a study cohort of the general population. The goal is to monitor this cohort for the long-term development of cardiac-, renal- and peripheral vascular end-stage disease. For that purpose the participants receive questionnaires on events and are seen every three/four years for a survey on cardiac-, renal- and peripheral vascular morbidity.  Website: http://www.prevend.org/index.php | This work was supported by the following grants: PREVEND genetics is supported by the Dutch Kidney Foundation (Grant E033), the National Institutes of Health (grant LM010098), The Netherlands Organization for Scientific Research (NWO-Groot 175.010.2007.006, NWO VENI grant 916.761.70, ZonMW 90.700.441), and the Dutch Inter University Cardiology Institute Netherlands. N. Verweij is supported by the Netherlands Heart Foundation (grant NHS2010B280). |  |
| --- | --- | --- | --- |
| Val Borbera (VB) | Val Borbera: The INGI‐Val Borbera population is a collection of 1,785 genotyped samples collected in the Val Borbera Valley, a geographically isolated valley located within the Appennine Mountains in Northwest Italy (1). The valley is inhabited by about 3,000 descendants from the original population, living in 7 villages along the valley and in the mountains. Participants were healthy people 18-102 years of age that had at least one grandfather living in the valley (2).  1. Traglia, M. et al. Heritability and demographic analyses in the large isolated population of Val Borbera suggest advantages in mapping complex traits genes. PLoS One 4, e7554 (2009).  2. Colonna V, et al. Small effective population size and genetic homogeneity in the Val Borbera isolate. Eur J Hum Genet. 2):89-94. 2013 | The research was supported by funds from Compagnia di San Paolo, Torino, Italy; Fondazione Cariplo, Italy and Ministry of Health, Ricerca Finalizzata 2008 and CCM 2010, PRIN 2009 and Telethon, Italy to DT. | We thank the inhabitants of the VB that made this study possible, the local administrations, the Tortona and Genova archdiocese and the ASL-22, Novi Ligure (Al) for support. We also thank Fiammetta Viganò for technical help, Corrado Masciullo and Massimiliano Cocca for building the analysis platform. |
