# Supplementary material for: Meta-GWAS and Meta-Analysis of Exome Array Studies Do Not Reveal Genetic Determinants of Serum Hepcidin
Source: PLoS One. 2016 Nov 15;11(11):e0166628. doi: 10.1371/journal.pone.0166628 (PMC5112847; doi:10.1371/journal.pone.0166628)
Supplement: S2 Table — (DOCX) [file pone.0166628.s002.docx]

**S2 Table.** Laboratory measurements.

| **Cohort** | **Time of blood sampling** | **Serum hepcidin** | **Serum ferritin** | **Serum iron** | **Transferrin or TIBC** | **TS** | **CRP** |
| --- | --- | --- | --- | --- | --- | --- | --- |
| NBS | Blood was sampled between between 8 AM and 9 PM; not fasting. | Serum hepcidin was measured in February 2010 in 2998 samples with an in house developed and validated competitive enzyme-linked immunosorbent assay as described before (Galesloot et al., Blood 2011; Kroot et al., Clin Chem 2010)). Detection limit: 0.18 nmoles/L (number of samples below detection limit=9). | Serum ferritin concentration was determined by a chemiluminescent microparticle immunoassay on the Abbott Architect calibrated against the ferritin assay on the Immulite 2000 of Diagnostic Products Corporation. | Colorimetric measurement using ascorbate/FerroZine reagents (Roche Diagnostics) on an Abbott Aeroset analyzer. | Unsaturated iron binding capacity was measured by adding a known quantity of Fe3+ to the serum samples, reducing it with ascorbate to Fe2+ and measuring it with FerroZine as described for total serum iron (Roche reagents on an Aeroset). TIBC was calculated by adding serum iron and unsaturated iron-binding capacity. | Serum transferrin saturation (TS)  was computed by dividing serum iron by TIBC. | CRP was quantified by immunologic agglutination detection with  latex-coupled polyclonal anti-CRP antibodies (Abbott Reagent on Aeroset). |
| PREVEND | Fasting blood samples in the morning | Hepcidin was measured in 6607 samples between October 2012 and March 2013 using the same assay as described for the NBS.  Detection limit: 0.5 nmoles/L (number of samples below detection limit=420). | Modular E170, Roche, Mannheim, Germany  Immunoassay (Sandwich)  Measuring range 0.50-2000 µg/L  LOD 0.50 µg/L | Colorimetric assay, Roche Modular P | Immunoturbidimetric assay, Roche Modular P | Sandwich immunoassay, Roche Modular E | Nephelometry (BNII N; Dade Behring, Marburg, Germany). |
| VB | Fasting blood samples (about 20 ml) were obtained in separate sessions, in the early morning after an overnight fast. | Serum hepcidin was measured with a validated mass spectrometry based method as described before (Traglia et al, J Med Genet 2011): surface enhanced laser desorption/ionisation time-of-flight mass spectrometry (SELDITOF-MS) using a PCS4000 (Bio-Rad, Hercules, California, USA) mass spectrometer, copper loaded immobilised metal affinity capture ProteinChip arrays (IMAC30-Cu2+), and a synthetic hepcidin analogue (hepcidin-24, Peptides International, Louisville, Kentucky, USA) as an internal standard [18], with recent technical improvements [19]. The lower limit of detection was  0.55 nM (number of samples below detection limit=175). | Serum ferritin concentration was determined using the following two instruments: HITACHI 917 ROCHE and Unicel Dx-C 800 BECKMAN | Serum iron concentration was determined using the following two instruments: HITACHI 917 ROCHE and Unicel Dx-C 800 BECKMAN | Transferrin was determined using the following two instruments: HITACHI 917 ROCHE and Unicel Dx-C 800 BECKMAN | Serum transferrin saturation (TS)  was computed by dividing serum iron by TIBC | CRP was quantified in in human serum by rate turbidimetry using High Sensitivity CRPH reagent, in conjunction with Beckman Coulter SYNCHRON LX®  PRO System, UniCel®  DxC 600/800  System(s) and SYNCHRON®  Systems CAL 5 Plus |
