# Supplementary material for: Meta-GWAS and Meta-Analysis of Exome Array Studies Do Not Reveal Genetic Determinants of Serum Hepcidin
Source: PLoS One. 2016 Nov 15;11(11):e0166628. doi: 10.1371/journal.pone.0166628 (PMC5112847; doi:10.1371/journal.pone.0166628)
Supplement: S3 Table — (DOCX) [file pone.0166628.s003.docx]

**S3 Table.** Phenotype information [median (5th percentile-95th percentile)] of the samples included in the meta-GWAS.

| **Discovery/**  **replication** | **Cohort** | **Sex** | **N** | **Age (years)** | **Hepcidin (nmoles/L)** | **Hepcidin/ferritin**  **(µmoles/µg)** | **Hepcidin/TS**  **(µmoles/L/%)** | **Ferritin**  **(µg/L)** | **Serum iron (µmoles/L)** | **TIBC**  **(µmoles/L)** | **TS (%)** | **CRP**  **(mg/L)** |
| --- | --- | --- | --- | --- | --- | --- | --- | --- | --- | --- | --- | --- |
| Discovery | NBS | M | 900 | 66  (55 – 77) | 8.5  (1.2 – 23.2) | 46.6  (17.1 – 120.6) | 0.3  (0.0 – 0.9) | 174.2  (29.5 – 532.5) | 18.0  (10.0 – 28.0) | 58.0  (45.0 – 73.0) | 30.8  (16.1 – 51.0) | <4  (<4 – 15) |
|  |  | F | 919 | 57  (39 – 74) | 6.7  (0.8 – 21.4) | 75.7  (29.7 – 203.1) | 0.2  (0.0 – 1.0) | 84.0  (12.4 – 266.7) | 16.0  (8.0 – 26.0) | 60.0  (47.0 – 77.0) | 26.6  (12.6 – 44.5) | <4  (<4 – 13) |
|  | PREVEND | M | 1495 | 50  (31 – 71) | 4.6  (0.9 – 10.7) | 30.7  (10.2 – 64.3 ) | 1.9  (0.3 – 4.6) | 179.6  (32.0 – 458.5) | 16.5  (9.0 – 27.0) | 63.2  (55.2 – 77.9) | 26.3  (13.8 – 42.7) | 1.2  (0.2 – 8.3) |
|  |  | F | 1407 | 48  (28 – 69) | 3.2  (0.3 – 8.2) | 45.9  (15.2 – 94.0) | 1.3  (0.1 – 3.4) | 87.0  (9.0 – 257.0) | 15.0  (7.0 – 25.0) | 66.7  (50.2 – 85.4) | 23.1  (9.2 – 39.8) | 1.3  (0.2 – 9.9) |
|  | VB | M | 688 | 57  (25 – 82) | 9.4  (2.5 – 29.6) | 71.7  (19.9 – 232.2) | 0.3  (0.1 – 1.0) | 134.0  (37.5 – 421.5) | 18.5  (10.4 – 29.7) | 58.5  (45.0 – 73.79) | 30.9  (17.2 – 53.3) | 1  (1 - 9) |
|  |  | F | 792 | 59  (26 – 82) | 6.9  (2.0 – 27.2) | 134.3  (37.9 – 589.6) | 0.3  (0.1 – 1.0) | 56.0  (11.0 – 171.7) | 16.5  (8.6 – 25.9) | 59.7  (47.2 – 80.7) | 27.0  (13.3 – 45.0) | 1  (1 - 9) |
| Replication | NBS | M | 457 | 53  (27 – 83) | 7.7  (1.4 – 20.0) | 50.2  (18.1 – 130.8) | 0.3  (0.1 – 0.9) | 147.7 (28.7 – 471.2) | 18.0  (9.0 – 30.1) | 59.0  (47.0 – 74.0) | 29.2  (15.0 – 50.9) | <4  (<4 – 11) |
|  |  | F | 540 | 55  (23 – 82) | 6.7  (0.7 – 20.1) | 75.8  (28.3 – 214.8) | 0.2  (0.0 – 0.9) | 79.9  (12.2 – 273.3) | 16.0  (8.0 – 26.0) | 61.0  (48.0 – 81.0) | 28.7  (12.0 – 45.9) | <4  (<4 – 13) |
|  | PREVEND | M | 1362 | 50  (31 – 71) | 4.3  (0.8 – 9.9) | 29.2  (9.9 – 63.0) | 1.8  (0.3 – 4.3) | 183.1  (33.0 – 458.0) | 16.8  (9.0 – 27.0) | 63.2  (50.2 – 77.9) | 27.0  (13.9 – 32.6) | 1.1  (0.2 – 9.1) |
|  |  | F | 1454 | 48  (31 – 69) | 3.2  (0.3 – 8.3) | 44.1  (13.8 – 93.6) | 1.3  (0.1 – 3.5) | 90.1  (9.0 – 262.0) | 15.2  (7.0 – 25.0) | 66.7  (52.7 – 87.9) | 23.3  (9.3 – 38.6) | 1.3  (0.2 – 9.0) |
