# Supplementary material for: Meta-GWAS and Meta-Analysis of Exome Array Studies Do Not Reveal Genetic Determinants of Serum Hepcidin
Source: PLoS One. 2016 Nov 15;11(11):e0166628. doi: 10.1371/journal.pone.0166628 (PMC5112847; doi:10.1371/journal.pone.0166628)
Supplement: S4 Table — (DOCX) [file pone.0166628.s004.docx]

**S4 Table.** Information about genotyping, imputation and quality control of the cohort-specific GWAS.

|  |  |  |  | **Imputation** | | |  |
| --- | --- | --- | --- | --- | --- | --- | --- |
| **Cohort** | **Genotyping platform** | **QC** | **N of clean SNVs and individuals** | **N of imputed SNVs** | **Reference panel** | **Software** | **Statistical analysis** |
| NBS | Illumina HumanHap370CNV-Duo BeadChip | Sample yield ≥96% (after exclusion of intensity-only markers (n=23,573)), Caucasian ancestry ≥89% (based on Structure analysis), SNV yield ≥96%, MAF ≥1%, and HWE p-value >10^-6^ | 1819 samples and 323,414 SNVs | 38,037,370 | 1000genomes phase1 integrated version 3 | IMPUTE2, pipeline see <http://www.bbmriwiki.nl/wiki/Impute2Pipeline> (Kanterakis et al., BMC Res Notes 2015). | SNPTEST v2.4.1 |
| PREVEND | Illumina Cyto SNP12 v2 | Population stratification was assessed by principal component analysis, Z-score > 3 for the first 5 principal components were excluded. Callrate <95%, duplicate samples and sex discrepancies were also excluded. Markers with call rate <95%, pHWE<0.00001, MAF ≥1% were included. | 3,649 samples and 232,571 SNVs | 12,862,598 | 1000genomes phase1 integrated version 3 | Minimac | SNPTEST v2.4.1 |

| VB | Illumina 370 Quad-CNV array, v3 | call rate >=90%, MAF >=1%, HWE p-value >10^-6^ | 1785 samples and 332,887 (for 1664 individuals with Illumina 370k chip);648,130 (for 121 individuals with Illumina OmniExpress 700k) | 38,043,574 | 1000genomes phase1 integrated version 3 | SHAPEITv2for 1664 individuals with Illumina 370K chip;none for 121 individuals with illumina OmniExpress 700k;  IMPUTE version 2.2.2 | R, GEMMA  SNPTEST  v2.4.1 |
| --- | --- | --- | --- | --- | --- | --- | --- |
