# Supplementary material for: Meta-GWAS and Meta-Analysis of Exome Array Studies Do Not Reveal Genetic Determinants of Serum Hepcidin
Source: PLoS One. 2016 Nov 15;11(11):e0166628. doi: 10.1371/journal.pone.0166628 (PMC5112847; doi:10.1371/journal.pone.0166628)
Supplement: S5 Table — (DOCX) [file pone.0166628.s005.docx]

**S5 Table.** Information about genotyping and quality control of the cohort-specific exome array analysis.

| **Cohort** | **Genotyping platform** | **Calling** | **QC** | **N of clean SNVs and individuals** | **Statistical analysis** |
| --- | --- | --- | --- | --- | --- |
| NBS | Illumina HumanExome-v1.1 | Data was called using the default genotype caller in Genome Studio. In addition, missing genotypes were recalled using the zCall algorithm, a genotype calling algorithm specifically designed for calling rare variants (Goldstein *et al.*, Bioinformatics 2012). | Sample QC was performed in data called in Genome Studio, samples were excluded from data that was also called with zCall. Marker QC was performed in the latter set.  Sample call rate>99%, exclusion of sex discrepancies, relatedness check (PI_HAT<0.25 included), heterozygosity (mean ± 4 SD included), population stratification (nearest neighbor 1-5 Z>-4 excluded), marker call rate>98%, HWE p>10^-6^. | 1,753 samples and 242,073 SNVs | Rvtests (program version: 20150629) |
| VB | Illumina HumanExome-12v1-2_A | Data was called using the default genotype caller in Genome Studio. In addition, missing genotypes were recalled using the zCall algorithm, a genotype calling algorithm specifically designed for calling rare variants (Goldstein *et al.*, Bioinformatics 2012). | Sample QC was performed in data called in Genome Studio, low quality samples were excluded before zCall. Filters applied: marker call rate≥95%, sample call rate≥95%, HWE p>10^-6^. | 1,782 samples and 243,959 SNPs | Rvtests (program version: 20140416) |
