# Supplementary material for: Meta-GWAS and Meta-Analysis of Exome Array Studies Do Not Reveal Genetic Determinants of Serum Hepcidin
Source: PLoS One. 2016 Nov 15;11(11):e0166628. doi: 10.1371/journal.pone.0166628 (PMC5112847; doi:10.1371/journal.pone.0166628)
Supplement: S6 Table — (DOCX) [file pone.0166628.s006.docx]

**S6 Table.** Phenotype information of the samples included in the exome array analyses [median (5th percentile-95th percentile)].*

| **Cohort** | **Sex** | **N** | **Age (years)** | **Hepcidin (nmoles/L)** | **Hepcidin/ferritin**  **(µmoles/µg)** | **Hepcidin/TS**  **(µmoles/L/%)** | **Ferritin**  **(µg/L)** | **Serum iron (µmoles/L)** | **TIBC**  **(µmoles/L)** | **TS (%)** | **CRP**  **(mg/L)** |
| --- | --- | --- | --- | --- | --- | --- | --- | --- | --- | --- | --- |
| NBS | M | 870 | 66  (55 – 77) | 8.5  (1.1 – 23.9) | 46.7  (17.2 – 121.1) | 0.26  (0.04 – 0.94) | 175.1  (29.9 – 529.1) | 18.0  (10.0 – 28.0) | 57.5  (45.0 – 73.0) | 30.8  (16.3 – 50.8) | <4  (<4 – 15) |
|  | F | 883 | 56 (39 – 74) | 6.7  (0.8 – 21.6) | 75.5  (30.4 – 204.1) | 0.24  (0.04 – 1.00) | 83.8  (12.5 – 266.5) | 16.0  (8.0 – 26.0) | 60.0  (47.0 – 77.0) | 26.6  (12.7 – 44.7) | <4  (<4 – 13) |
| VB | M | 686 | 57  (25 - 82) | 9.4  (2.5-29.6) | 72.1  (19.9 – 232.3) | 0.30  (0.08 – 0.97) | 134.0  (37.3 – 422.5) | 18.5  (10.4- 29.7) | 58.5  (45.0 – 73.8) | 30.9  (17.2– 53.3) | 1  (1 -9) |
|  | F | 788 | 59  (26 – 84) | 6.9  (2.0 -27.1) | 134.3  (37.8 – 593.9) | 0.26  (0.08 – 0.96) | 56.0  (11 .0– 171.9) | 16.5  (8.6 – 25.9) | 59.7  (47.2 – 80.8) | 27.0  (13.3 – 45.0) | 1  (1 -9) |

*For both NBS and VB, samples do largely overlap with but are not completely the same as the samples included in the GWAS analyses (Supplemental Table 3).
