# Supplementary figures and images for: Meta-GWAS and Meta-Analysis of Exome Array Studies Do Not Reveal Genetic Determinants of Serum Hepcidin
Source: PLoS One. 2016 Nov 15;11(11):e0166628. doi: 10.1371/journal.pone.0166628 (PMC5112847; doi:10.1371/journal.pone.0166628)

**
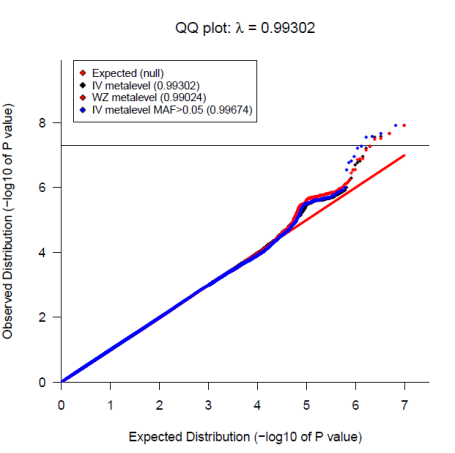
S2 Figure.** Manhattan plot and QQ plot for the meta-analysis
results for hepcidin in the subset.


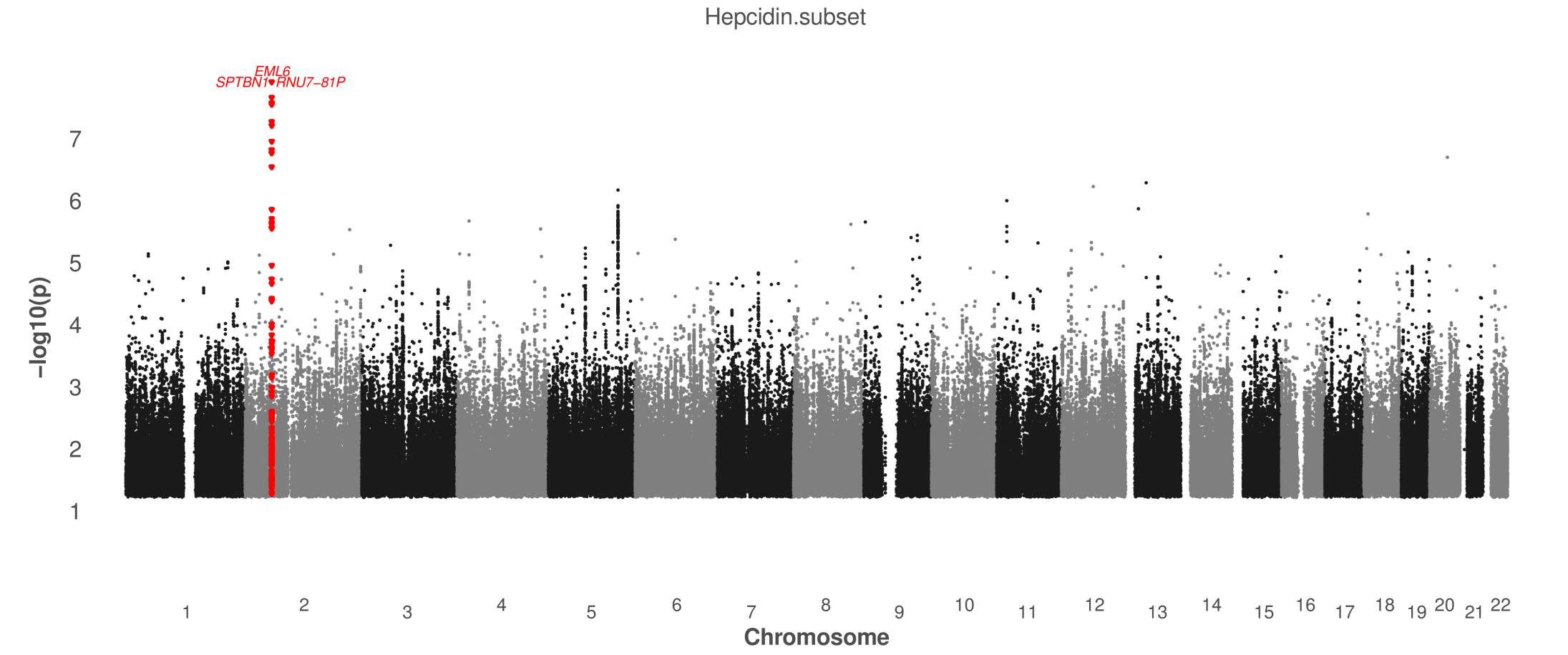

Supplement: S2 Fig — (DOCX) [file pone.0166628.s015.docx]

**S3 Figure.** Regional association plot for rs118031191 with serum hepcidin in all individuals.
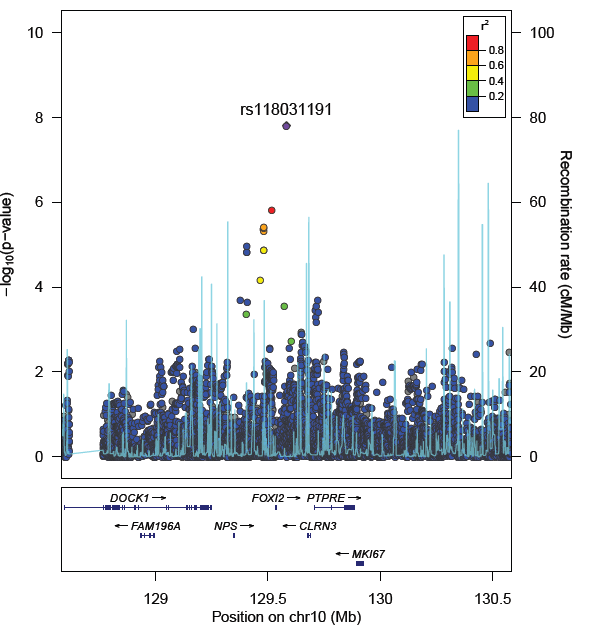

Supplement: S3 Fig — (DOCX) [file pone.0166628.s016.docx]

**S4 Figure.** Regional association plot for rs354202 with serum hepcidin in the subset.


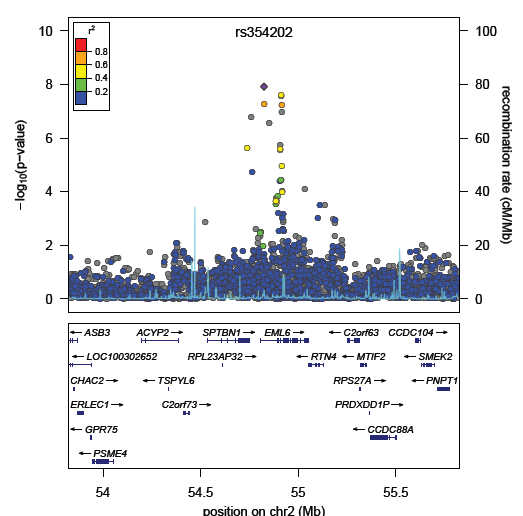

Supplement: S4 Fig — (DOCX) [file pone.0166628.s017.docx]
