# Supplementary material for: Meta-GWAS and Meta-Analysis of Exome Array Studies Do Not Reveal Genetic Determinants of Serum Hepcidin
Source: PLoS One. 2016 Nov 15;11(11):e0166628. doi: 10.1371/journal.pone.0166628 (PMC5112847; doi:10.1371/journal.pone.0166628)
Supplement: S5 Fig — (DOCX) [file pone.0166628.s018.docx]

**S5 Figure.** Regional association plot for the chromosome 2 locus with serum hepcidin conditioned on rs354202 in all individuals (NBS data only).

**
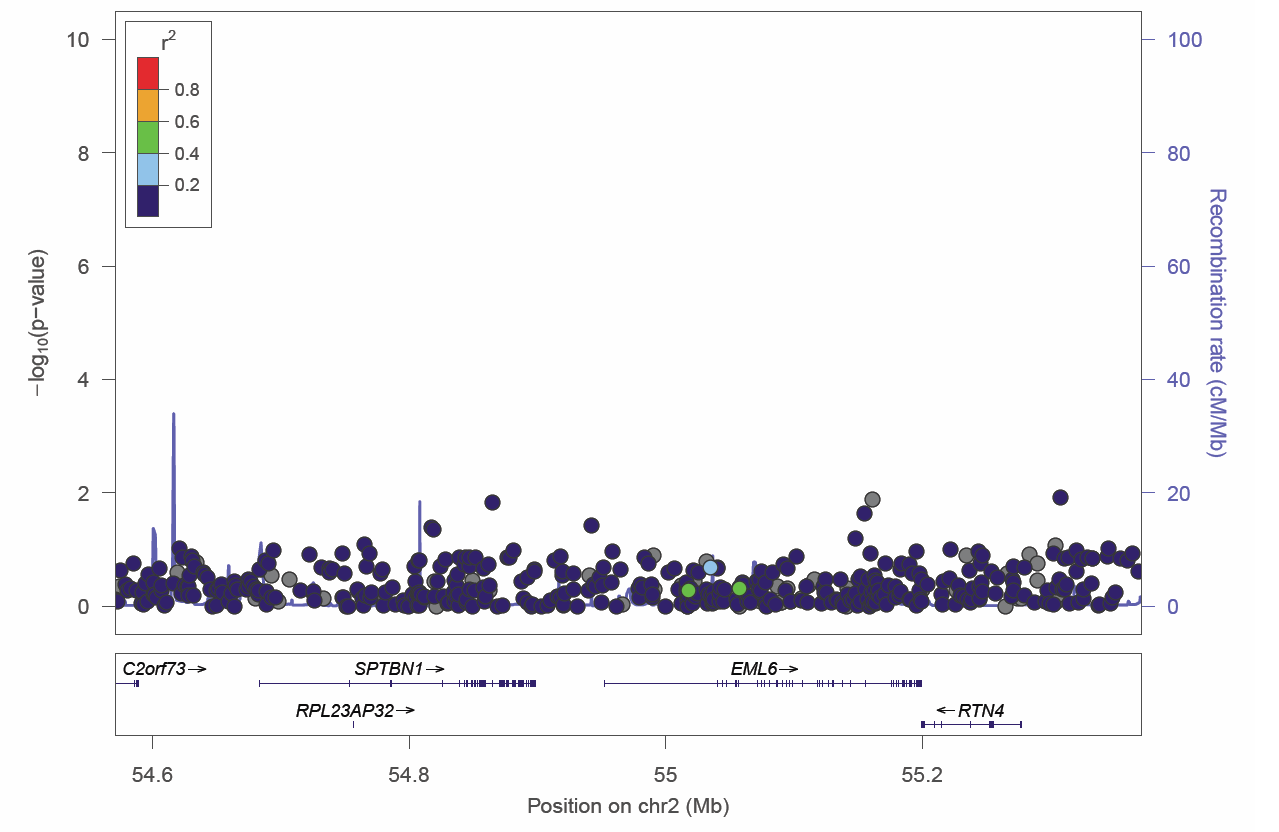
**
