# Supplementary material for: Meta-GWAS and Meta-Analysis of Exome Array Studies Do Not Reveal Genetic Determinants of Serum Hepcidin
Source: PLoS One. 2016 Nov 15;11(11):e0166628. doi: 10.1371/journal.pone.0166628 (PMC5112847; doi:10.1371/journal.pone.0166628)

**
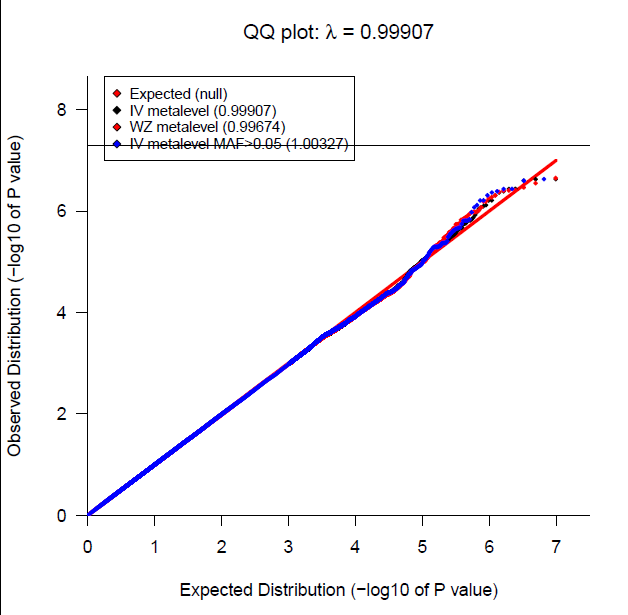
S9 Figure.** Manhattan plot and QQ plot for the meta-analysis
results for the ratio hepcidin/TS in all individuals.


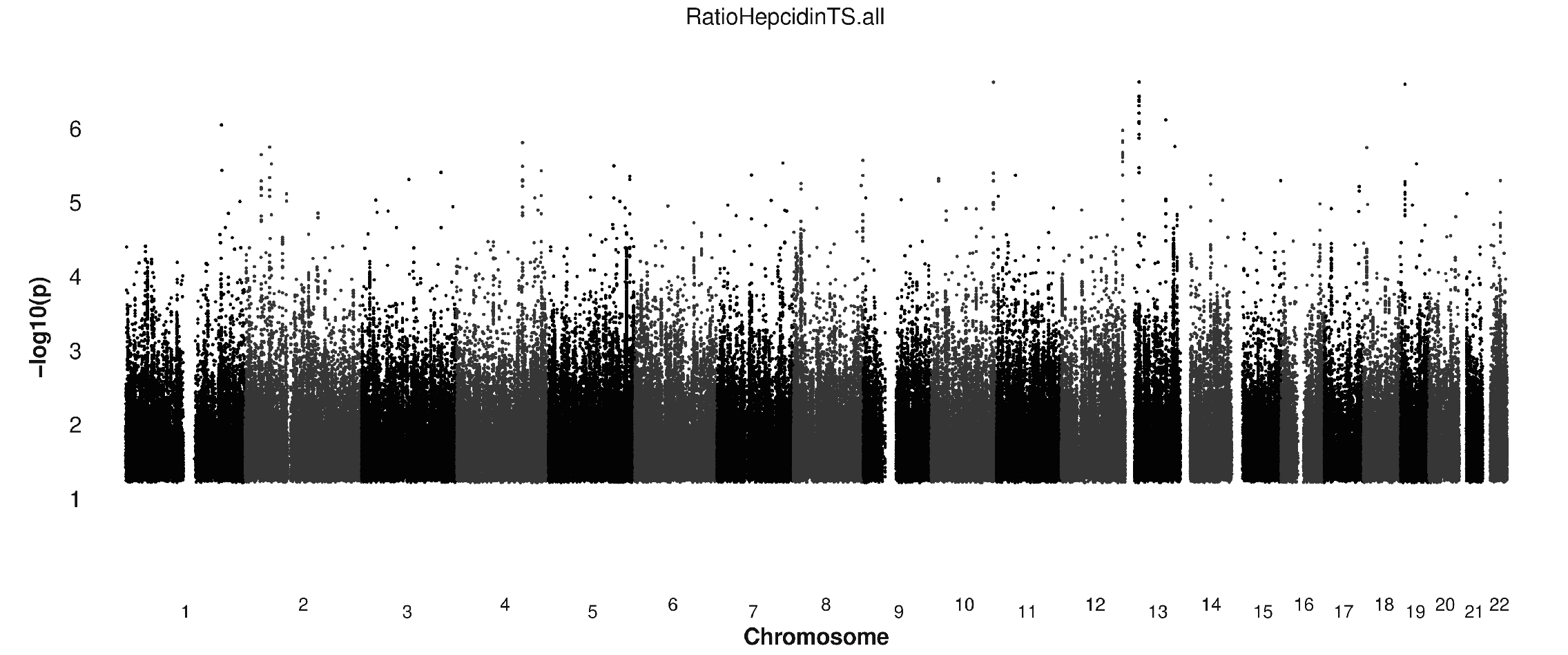

Supplement: S9 Fig — (DOCX) [file pone.0166628.s022.docx]
