# Supplementary material for: Meta-GWAS and Meta-Analysis of Exome Array Studies Do Not Reveal Genetic Determinants of Serum Hepcidin
Source: PLoS One. 2016 Nov 15;11(11):e0166628. doi: 10.1371/journal.pone.0166628 (PMC5112847; doi:10.1371/journal.pone.0166628)
Supplement: S1 File — (DOCX) [file pone.0166628.s024.docx]

**S1 File: Supplemental Methods**

Data-driven Expression-Prioritized Integration for Complex Traits (DEPICT)

DEPICT systematically identifies the most likely causal gene at a given associated locus, tests gene sets for enrichment in associated SNPs, and identifies tissues and cell types in which genes from associated loci are highly expressed (see Pers et al., 2015 for a detailed description of the method). First, DEPICT assigns genes to associated SNPs using LD r2 > 0.5 distance to define locus boundaries, merges overlapping loci and discards loci mapping within the extended major histocompatibility complex region (chromosome 6, base pairs 25,000 – 35,000). Next, DEPICT prioritizes genes within the associated loci based on genes functional similarity to genes from other associated loci within the same GWAS (genes that are similar to genes from other loci obtain low prioritization P values), and adjusts for gene length bias as well as other potential confounders by use of simulated GWAS results. There can be several prioritized genes in a given locus. Next, DEPICT conducts gene set enrichment analysis by testing whether genes in associated loci are enriching for reconstituted versions of known biological pathways, gene sets as well as protein complexes. Leveraging the guilt by association hypothesis that genes co-expressing with genes from a given gene set are likely to be part of that gene set (See Cvejic et al.57 for details), the gene set reconstitution is accomplished by identifying genes that were co-expressed with genes in a given gene set based on a panel of 77,840 gene expression microarrays. Gene sets from the following repositories were reconstituted: 5,984 protein complexes originating from 169,810 high-confidence experimentally-derived protein-protein interactions58; 2,473 phenotypic gene sets derived from 211,882 gene-phenotype pairs from the Mouse Genetics Initiative59; 737 Reactome database pathways60; 184 KEGG database pathways61; and 5,083 Gene Ontology database terms62. Finally, DEPICT conducts tissue and cell type enrichment analysis, by testing whether genes in associated loci are highly expressed in microarray-based gene expression data covering 209 Medical Subject Heading annotations (37,427 Affymetrix U133 Plus 2.0 Array samples are used for this analysis). See Wood et al., Geller et al. and van der Valk et al. for previous applications of DEPICT. For this work we first used the PLINK software tool to clump all SNPs with association P value < 1 x 10-5 as input (parameters: ‘--clump-p1 1e-5 --clump-kb 500 --clump-r2 0.05’). Gene set enrichment and tissue/cell type enrichment analyses were run using the default settings in DEPICT.
